# Infrastructure for bioinformatics applications in Tanzania: Lessons from the Sickle Cell Programme

**DOI:** 10.1371/journal.pcbi.1010848

**Published:** 2023-02-23

**Authors:** Liberata A. Mwita, William F. Mawalla, Frank R. Mtiiye, Daniel Kandonga, Jill Kent, Julie Makani, Raphael Z. Sangeda

**Affiliations:** 1 Department of Pharmaceutical Microbiology, Muhimbili University of Health and Allied Sciences, Dar es Salaam, Tanzania; 2 Department of Haematology and Blood Transfusion, Muhimbili University of Health and Allied Sciences, Dar es Salaam, Tanzania; SIB Swiss Institute of Bioinformatics, SWITZERLAND

## Abstract

Sickle cell disease (SCD) is a common genetic disorder in Africa. Some ongoing work in SCD research includes the analysis and comparisons of variation in phenotypic presentations and disease outcomes with the genotypic signatures. This has contributed to the observed growth of molecular and genetic data in SCD. However, while the “omics” data continues to pile, the capacity to interpret and turn the genetic findings into clinical practice is still underdeveloped, especially in the developing region. Building bioinformatics infrastructure and capacity in the region is key to bridging the gap. This paper seeks to illustrate how the Sickle Cell Programme (SCP) at the Muhimbili University of Health and Allied Sciences (MUHAS) in Tanzania, modeled the integration of infrastructure for bioinformatics and clinical research while running day-to-day clinical care for SCD in Tanzania.

## Background

Bioinformatics is an interdisciplinary application of computation tools to harvest, organize, analyze, link, and store biological molecular data [1]. Clinical and epidemiological research continues to reveal significant phenotypic differences in presentation, progression, and disease outcomes in cohorts of patients such as those with sickle cell anemia, who were initially thought to have a common disease etiology [2,3]. The diverse presentations and clinical manifestations in these patients necessitate the need for genetics and bioinformatics research to elucidate the underlying causes of variations to improve individual patient management [2,4]. Thus, genome-wide association studies (GWAS) are expected to play a key role in the understanding of the genetic and molecular mechanisms underlying individual disease presentation [5,6]. GWAS has benefited sickle cell disease (SCD) research by helping to identify variants that are associated with SCD clinical complications such as vaso-occlusive pain and stroke [7–11]. In addition, GWAS results are now used to input data when doing functional studies to reveal mechanisms of candidate genes/single nucleotide polymorphisms (SNPs).

### Bioinformatics and sickle cell disease

SCD is the most common monogenic disease in humans [12]. Variation in hemoglobin S (HbS) occurs due to a single nucleotide substitution GAG→GTG in codon 6 of the beta-globin gene (E6V) on chromosome 11p15.5, resulting in a change of amino acid from glutamic acid to valine. On the other hand, mutation of the same codon in the hemoglobin C results in GAG→AAG change and substitution of amino acid from glutamic acid to lysine (E6K). Individuals with sickle cell anemia may have HbSS or HbSC that are the homozygous genotypes from variation in hemoglobin S and hemoglobin C while individuals with sickle cell trait may have HbAS or HbAC genotypes [13–16].

About 75% of the affected populations are born in Africa [12]. The disease follows the malaria belt due to the protection against *P*. *falciparum* malaria for those who are heterozygotes for the variant (HbAS) and therefore carriers of the disease [15,16]. Efforts to answer the question of how the β^s^–gene originated and spread in the malarial belt region lead to the identification of haplotypes in β^s^–gene cluster using restriction endonucleases [17]. Even though the resulting molecular defect at the β globin protein level is the same across the haplotypes, clinical manifestation, disease history, and severity vary significantly across patients [18,19]. The most prominent haplotypes in Africa are Cameroon in Central Africa, Senegal and Benin in West Africa, and Bantu (also known as the Central Africa Republic haplotype) in East and Central Africa. Early studies showed the Bantu haplotype to be associated with the worst prognosis, with an increased risk of development of complications and early mortality [14,20]. In particular, the Bantu haplotype is linked with severe vaso-occlusive events, stroke, early-onset of end-organ failure (i.e., lung, kidney, and eyes), and death, compared with non-Bantu haplotypes [20]. Until recently, the β^s^-gene was thought to arise from different mutations occurring at different times in the same locus, explaining the existence of different haplotypes. However, a more recent analysis supports the theory of single-origin mutation that occurred before the development of haplotypes [21].

The Sickle Cell Programme (SCP) [22] is one of the Tanzanian H3ABioNet nodes. The H3AbioNet supports the bioinformatics capacity and the team working on the data that was collected by clinicians and researchers in the different research projects at SCP [23].

The SCP began with a database of GWAS data of 1,952 SCD individuals that originally sought to elucidate the association between SCD and fetal hemoglobin (HbF) [11,22]. The findings of the original study led to a new study that selected a few individuals with extreme HbF to identify genetic variants associated with extreme fetal hemoglobin levels using targeted next-generation sequencing [24]. The GWAS database has now enabled the initiation of several other parallel studies. The SCP maintains a server dedicated to bioinformatics analysis. It also has access to high-performance computing (HPC) through collaboration with the Dar-es-salaam Institute of Technology (DIT) in Tanzania that provides opportunities for computational resources. At the moment, there are 2 main bioinformatics pipelines that the network supports: GWAS data analysis and next-generation sequencing (NGS) data analysis. The center has GWAS data of approximately 2,000 patients. There is also a continuous analysis of different phenotypes/conditions (i.e., hemoglobin F levels, anemia, and liver function) with the genotypes of the patient. The team has 2 bioinformaticians, 1 software engineer, 1 informatician, and 1 statistician employed to support the bioinformatics infrastructure. The team works closely within the program and program members include data clerks, clinicians, nurses, and other researchers.

### Bioinformatics in Africa

As a discipline and an integral component in health research and disease causal-intervention analysis, bioinformatics is lagging in Africa [25]. To build bioinformatics capacity and familiarize the continent and its scientists in this growing field, the Human Heredity and Health in Africa (H3Africa) (https://h3abionet.org/) initiative provides a framework for integration and communication to enable full exploitation of Africa’s genomic and environmental data [26]. H3AbioNet, the Pan African Bioinformatics Network is building bioinformatics capacity through different tasks, including the building of Pan-African informatics infrastructure and providing informatics support to H3Africa projects, in addition to developing the H3Africa data coordinating center [27,28]. It also organizes regular online and onsite short bioinformatics courses in several African countries to enable participants to acquire basic and advanced knowledge of bioinformatics [29]. Some African Universities that received H3ABioNet funding were equipped with computational facilities such as desktops and servers that enabled them to have dedicated computer laboratories for running bioinformatics practical classes. This allowed the commencement of undergraduate bioinformatics courses in these universities [27,30].

In South Africa, bioinformatics started in the mid-1990s with the founding of the South Africa National Bioinformatics Institute (SANBI) at the University of Western Cape [31]. SANBI expanded throughout the country by establishing computational biology units in many universities involved in undergraduate and postgraduate training in bioinformatics and research activities. Researchers involved the government from the beginning; the latter provides funds and creates employment opportunities [31]. The establishment of societies, networks, and collaboration within and outside the country contributed to the rapid growth of the bioinformatics field [31,32]. South Africa is considered a leader in bioinformatics in African countries [31].

In the early 2000s, the West African countries led by Nigeria and Ghana started introducing bioinformatics as an academic field by hosting seminars, workshops, and symposia and later developing postgraduate courses [32–35]. The growing demand for experts and bioinformatics applications led to establishment of the Nigerian Bioinformatics and Genomics Network (NBGN) in 2019 [34].

Elsewhere, in Eastern Africa, the support from Fogarty International Center of the National Institutes of Health (FIC-NIH, United States of America) and the Initiative to Develop African Research Leaders (IDeAL) facilitated the starting of the Eastern Africa Network for Bioinformatics Training (EANBiT). EANBiT (http://eanbit.icipe.org/) is a network of universities and research centers in Kenya, Uganda, and Tanzania. It offers fellowships for bioinformatics training at the Master’s level to increase the number and quality of potential PhD students and researchers.

### Bioinformatics in other countries

Different countries have followed different paths in recognizing the bioinformatics needs, building capacity in terms of infrastructures, and education to necessitate the start and continuation of bioinformatics in their settings. Greece and Cyprus [36] started their bioinformatics journey in the 1980s, they took 20 years of establishment, collaboration was key to recognition of bioinformatics activities in both countries, and informal and formal meetings between research groups were crucial. The establishment was followed by a recognition stage that led to the growth of bioinformatics in terms of research activities, education and training, service provision, and other community and infrastructure projects. They recommended the identification of national priorities, expanding research and research support without forgetting training at all levels and connecting with industries, and participation in local and international conferences as some of the means that facilitated the growth of bioinformatics in the 2 countries [36]. The objectives of bioinformatics training differ in the level of details and contents depending on the needs of the end user. Bioinformatics training is crucial as data size and complexity of sequencing data increases, there is a need to tailor the training and education according to the needs of the end user [37]. Different countries with different backgrounds, tools, and knowledge should tailor their bioinformatics training and research needs accordingly.

### The integration model of sickle cell disease research in Tanzania

**Fig 1:**
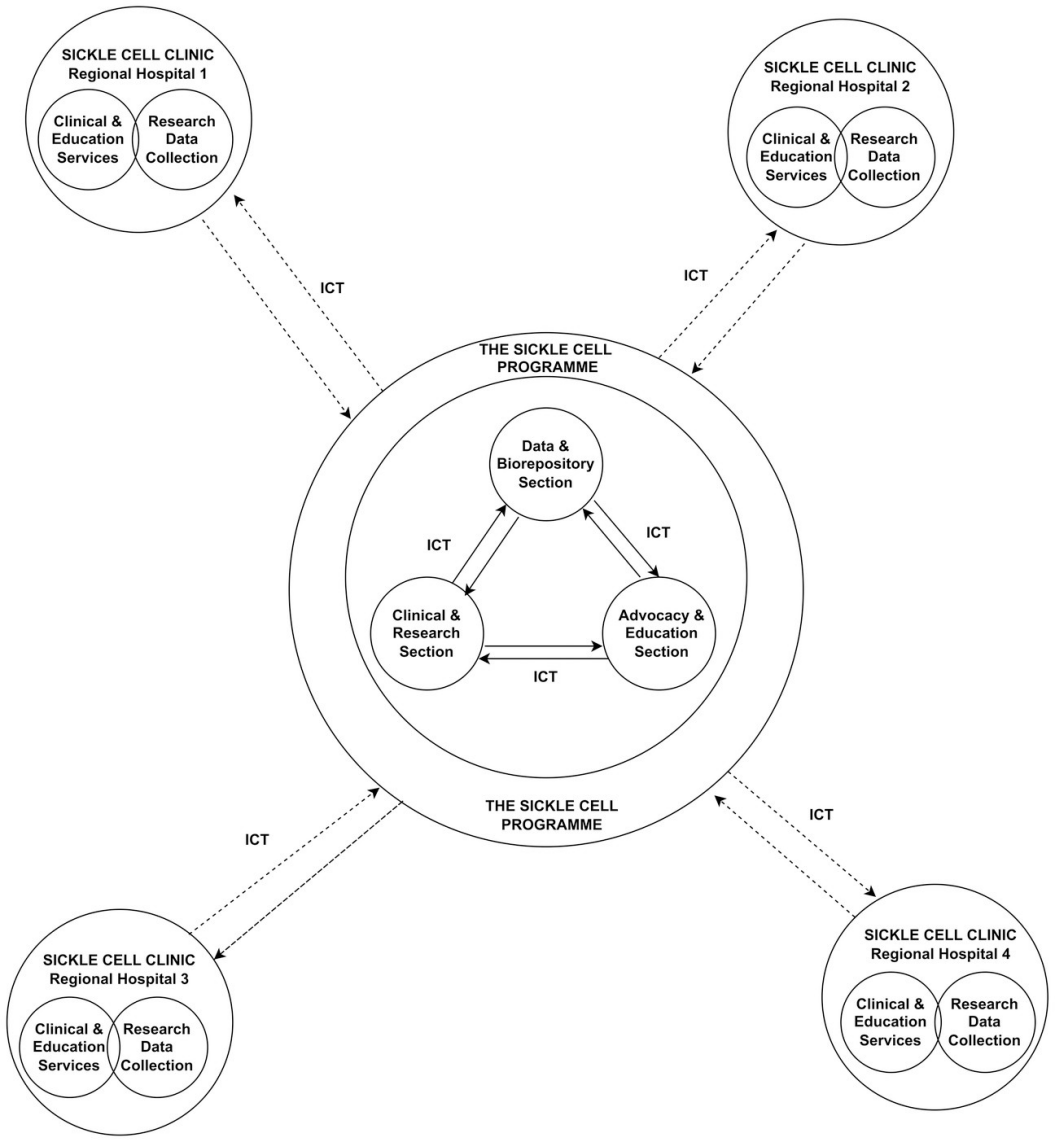
SCP service integration model: The SCP has a central office that coordinates all the program’s activities. The center has 3 key sections; the Clinical and Research unit, the Data and Biorepository unit, and the Advocacy and Education unit. The center supports and coordinates the activities of the sickle cell clinics across the regional hospitals, and all the clinics are linked with the center’s servers using ICT systems, enabling online data collection and communication.

The SCP engages in health provision in parallel with research, education, and advocacy activities [38]. Comprehensive healthcare is made possible through the integration of data capture and management, ICT, and day-to-day clinical services. The approach provides a platform for improved clinical care, research, and rapid feedback mechanisms (Fig 1).

### Sickle cell disease clinic operations

The SCP has been providing healthcare to SCD patients for 16 years, since its inception [22]. It started with enrolling patients attending MNH who were known or suspected to have SCD and their invited relatives and began with 2 clinics per week. At the moment, the SCP operates weekly SCD clinics at MNH (1 pediatric and 1 adult clinic), Mwananyamala Regional Hospital (1 combined pediatric and adult clinic), Temeke Regional Hospital (1 combined pediatric and adult clinic), Amana Regional Hospital (1 combined pediatric and adult clinic), and MNH-Mloganzila (1 combined pediatric and adult clinic). The SCP also runs a newborn screening program at Temeke and Mwananyamal Hospitals. Plans are underway to expand clinics and newborn screening centers to all tertiary-level hospitals in the country [39].

The sickle cell clinics have 2 primary activities: (1) clinical and education provision; and (2) research data collection. These activities are jointly facilitated by respective units of the SCP [22].

For research purposes, individuals who are confirmed with SCA (HbSS or HbSβ^0^) are first consented before being enrolled in the sickle cell database. Patients are informed about any studies or research projects that are ongoing and asked if they wish to take part. If interested, they give written consent before they take part in any study. Depending on the research project, community, clinical, laboratory, or radiological data are collected and transferred to the Sickle Cell Programme Data and Biorepository section for analysis and/or storage. The SCP also collects and stores biological samples for genomic studies from patients who have participated in different SCD research studies.

## Data and biorepository

The SCP data and biorepository section was established as a unit in 2004 to enable data collection and management of data generated from past and ongoing studies. The SCP has now accumulated a wealth of longitudinal phenotypic, genotypic, and biological data from the 18 years of data collection. The data and biorepository section is responsible for secure data capture and input, organization, presentation and sharing, storage and maintenance of the SCP data. It designs data capture tools and health passports in collaboration with clinicians. These are used by clinicians and researchers at sickle cell clinics in regional hospitals. The data collected from hospitals contains the medical record number (MRN) for validation and verification, which serves as the unique file number of the patient. This ensures patient information is accurate, complete, and timeless. The program uses the built-in function in Research Electronic Data Capture (REDCap) for exchanging data between systems using application program interface (API) that also supports HL7 protocols, this allows data to be imported and exported to other electronic health record systems including health information systems. Pfsense firewall (a combination of hardware and software) is part of a broader cybersecurity strategy for the program, which has been designed to protect program infrastructure from outside threats such as viruses, malware, spyware, and other malicious software. The firewall has been built also to prevent unauthorized access to the program’s internal network resources. Moreover, the Pfsense firewall is used to limit bandwidth usage for certain types of data. Lastly but not the least, Pfsense is used to provide VPN services that use encryption technology to secure communications between 2 parties. The encrypted traffic travels across public networks like the internet without anyone else knowing about it.

Clinicians and researchers use case report forms (CRFs) as tools to collect clinical and biological data. The data collected is sent to the SCP for storage and data entry. After validation, the data is recorded in the web-based database and the biological samples are sent to the biorepository bank for future analysis. Another critical function of the data section is the facilitation of efficient retrieval of data for analysis and undertaking active recruitment and follow-up of patients and research subjects. Standard operating procedures (SOPs) are well implemented to guide the process of data collection. Training is conducted for data collectors regularly and during procedural changes. The program uses barcode scanners (health passports) for quality checks to manage data collected from hospitals. A set of identifiers are used in line with built-in function rules that are applied during data entry into the repository. The country number is generated with a barcode to uniquely identify 1 patient before the other and the record number (MRN) as a hospital identifier was employed for easier searching and retrieval of patient information.

As part of the Sickle in Africa Consortium member, the SCP collaborates with Sickle Africa Data Coordinating Center (SADaCC) through the Sickle Pan-Africa Research Consortium (SPARCO) project for data elements standardization and harmonization at the consortium level (https://www.sickleinafrica.org), which will then ensure the quality of data collected among participating countries for future collaborative research.

To achieve the aforementioned tasks, the data collection tools, including electronic tablets, or physical health passports are assigned with a barcode and linked to an electronic health record system. REDCap software, the SCP’s main database software [40,41] is used for data capturing, storage, and maintenance. Data stored in the Redcap Database is secured through pfSense (Open Source Firewall). In addition to soft data, the SCP maintains archived samples of DNA, red blood cells, plasma, and serum from over 5,000 SCD patients in its biorepository bank.

**Fig 2:**
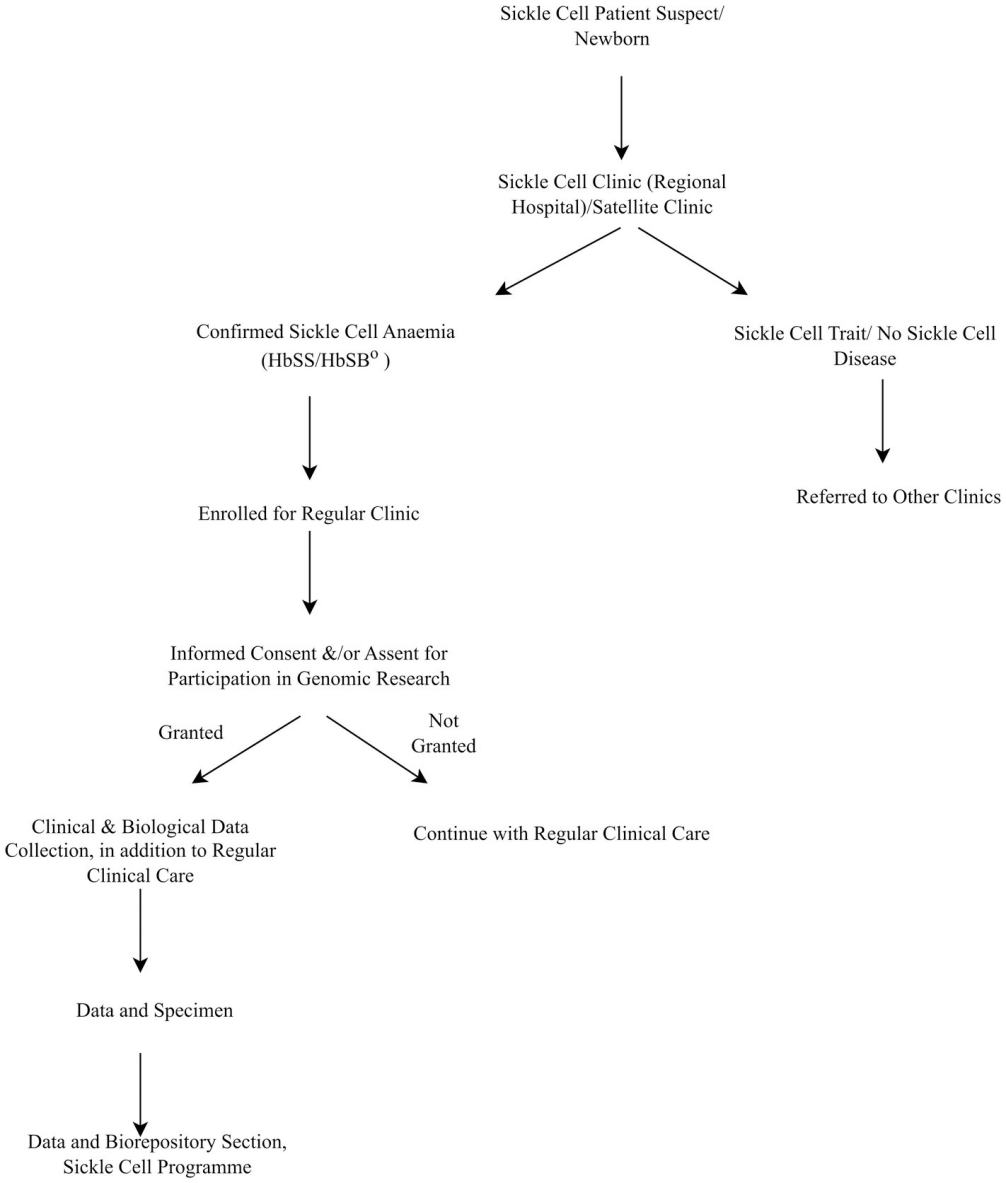
Data flow from satellite sickle cell clinics to the data and biorepository section.

The data section performs weekly data backup and storage to the file server located at the SCP. The SCP uses computational database triggers to keep track of any changes committed to the database as soon as data is entered. A networked infrastructure enables data to be saved to the local servers within the MUHAS campus. Data collection and data recovery plans in case of failure are undertaken according to locally established SOPs. Data collection and data recovery plans in case of failure are undertaken according to locally established SOPs that govern the data collection, management, and recovery processes for data protection and security of CRFs and database servers. Fig 2 shows how the data flows from clinical to data and the biorepository section.

## Information and communication technology (ICT)

The SCP ICT section started as a small unit supporting the SCP center. In 2006, the section expanded to provide ICT services to 3 sickle cell clinics at MNH, connecting them to the SCP. Currently, the section has expanded and integrated a network of satellite clinics at regional hospitals in the Dar es Salaam region.

The SCP has a contract agreement with Tanzania Education and Research Network (TERNET) (https://www.ternet.or.tz/), a provider of a high-speed fiber network link with a connection speed of 6 Mbps uplink and 6 Mbps downlink which is backed up by a wireless link. This network system powers the connection and integration of various activities at the SCP. The SCP ICT section maintains the SCP’s ICT infrastructure, local area network (LAN), and internet connectivity. It also designs, develops, and supports platforms for secure data transfer, online meeting/teleconference, task manager/helpdesk systems, and maintenance of the SCP’s hardware. The section prepares SOPs and guidelines for SCP’s systems, tools, and hardware.

## Future of bioinformatics in Tanzania

MUHAS is a member of H3BioNet. In recent years, the University of Dar es Salaam, MUHAS, and Sokoine University (SUA) have increased bioinformatics staff and are serving as hubs for genetic training and research. Plans are underway to establish advanced-level training programs (Masters in science and PhD in bioinformatics). Local capacity building, regional and global collaboration, and increased investment in bioinformatics infrastructure will pave the way for advanced genomic research projects in Tanzania.

The SCP can serve as a model for research programs and institutions seeking to develop and apply bioinformatics. The SCP model is particularly practical in the developing region where resources are constrained and research institutions and programs are still in the developmental stages.

## Recommendations

To incorporate bioinformatics in a research program, personnel and infrastructures are needed, those are key regardless of limited settings. The assumption is, a colleague who wants to implement the model already has a research idea and or data that requires bioinformatics expertise. First, there is a need to hire a bioinformatician (s) or train the researchers you have (with a background either in molecular biology or information technology). If training the personnel you have is the best approach, they can take a series of short courses in bioinformatics starting from introduction to bioinformatics or enroll in a postgraduate program (MSc, PhD in bioinformatics). Apart from bioinformaticians, a colleague will need a team of other experts. In our setting, the team include data clerks, data managers, clinicians, bioinformaticians, statisticians, and informaticians. The infrastructure needed includes computers, a good internet connection, a server, and software for analysis.

The availability of the team who work tirelessly, patients’ availability to participate in the research, timely ICT assistance, availability of the internet and good internet speed, and availability of electricity are needed to support the program and the infrastructure.

There is a need to continuously look for funding to support the program and keep personnel and the infrastructure running. The other challenge is to get and keep bioinformaticians in the program because there may be few, hence if a colleague is in similar settings, he/she has to establish training programs.
